# Host genetic variation drives the differentiation in the ecological role of the native *Miscanthus* root-associated microbiome

**DOI:** 10.1186/s40168-023-01646-3

**Published:** 2023-09-30

**Authors:** Niuniu Ji, Di Liang, Lindsay V. Clark, Erik J. Sacks, Angela D. Kent

**Affiliations:** 1 DOE Center for Advanced Bioenergy and Bioproducts Innovation, University of Illinois at Urbana-Champaign, Urbana, IL 61801 USA; 2 Institute for Sustainability, Energy and Environment, University of Illinois at Urbana-Champaign, Urbana, IL 61801 USA; 3 Department of Crop Sciences, University of Illinois at Urbana-Champaign, Urbana, IL 61801 USA; 4 Department of Natural Resources and Environmental Sciences, University of Illinois at Urbana-Champaign, Urbana, IL 61801 USA

**Keywords:** Perennial plant microbiomes, Rhizosphere soil, Microbiome assembly, Microbial co-occurrence networks, Core microbiome, Host genetic variation

## Abstract

**Background:**

Microbiome recruitment is influenced by plant host, but how host plant impacts the assembly, functions, and interactions of perennial plant root microbiomes is poorly understood. Here we examined prokaryotic and fungal communities between rhizosphere soils and the root endophytic compartment in two native *Miscanthus* species (*Miscanthus sinensis* and *Miscanthus floridulus*) of Taiwan and further explored the roles of host plant on root-associated microbiomes.

**Results:**

Our results suggest that host plant genetic variation, edaphic factors, and site had effects on the root endophytic and rhizosphere soil microbial community compositions in both *Miscanthus sinensis* and *Miscanthus floridulus*, with a greater effect of plant genetic variation observed for the root endophytic communities. Host plant genetic variation also exerted a stronger effect on core prokaryotic communities than on non-core prokaryotic communities in each microhabitat of two *Miscanthus* species. From rhizosphere soils to root endophytes, prokaryotic co-occurrence network stability increased, but fungal co-occurrence network stability decreased. Furthermore, we found root endophytic microbial communities in two *Miscanthus* species were more strongly driven by deterministic processes rather than stochastic processes. Root-enriched prokaryotic OTUs belong to Gammaproteobacteria, Alphaproteobacteria, Betaproteobacteria, Sphingobacteriia, and [Saprospirae] both in two *Miscanthus* species, while prokaryotic taxa enriched in the rhizosphere soil are widely distributed among different phyla.

**Conclusions:**

We provide empirical evidence that host genetic variation plays important roles in root-associated microbiome in *Miscanthus*. The results of this study have implications for future bioenergy crop management by providing baseline data to inform translational research to harness the plant microbiome to sustainably increase agriculture productivity.

Video Abstract

**Supplementary Information:**

The online version contains supplementary material available at 10.1186/s40168-023-01646-3.

## Background

The plant rhizosphere harbors complex microbial communities, which play vital roles in the health and development of host plants [1–3]. Plants regulate root-associated microbes through diverse mechanisms [4]. First, through plant signals and root exudates, plants can directly recruit rhizosphere microbiomes by selecting microbes that can penetrate root tissues to form endophytes [5]. Plants could also alter rhizosphere soil physical and chemical properties to indirectly influence root-associated microbes [6]. Many of these mechanisms are dictated by the host genotype; however, the relative importance of host plant genetic variation versus soil conditions in regulating root-associated microbial community remains unclear.

Within a plant species, host genetic variation influences the composition of rhizosphere microbiota. For example, maize genotype has been shown to explain a significant fraction of heritable variation in rhizosphere microbial diversity [7, 8]. It was also demonstrated that key plant loci, instead of the whole plant genome, contribute to the establishment of plant microbiome through plant functional genes [9–11]. Additionally, host plants can employ a range of gene pathways such as nutrient uptake and transport to alter soil nutrient solubility to promote the colonization of microbes [12]. Importantly, the study of molecular mechanisms of plant-microbiome interactions is still in the early stages, and how the plant genetic networks regulate microbial community composition remains unclear. Understanding how host plant genetic variation affects the microbiome will be a crucial step toward harnessing the microbiome for agricultural productivity [9], but directing the microbiome towards more sustainable assemblages will be particularly important as we develop new crop cultivars.

Plant microbiomes are highly diverse, yet not all of these microorganisms have important functions in their host. The core microbiome, which is a set of microbial taxa that are found in most samples of a particular set of plants, is considered a key component for organizing the assembly of plant-associated microbiomes and promoting host plant growth [13]. Core microbes interact with other microbial taxa via cooperation and competition, which also play major ecological roles in maintaining the complex microbial networks and in driving belowground nutrient cycling and functional stability of soil microbiomes [14, 15]. Despite the extensive efforts on identifying the core microbial members [16, 17], knowledge gaps remain in determining how host plant genetic variation affects the core microbial community and microbial co-occurrence network [13].

To better understand how plant genetic variation affects rhizosphere microbiomes, we investigated the endophytic and soil microbial communities (prokaryote and fungi) in roots and rhizosphere of *Miscanthus sinensis* and *Miscanthus floridulus*. *M. sinensis* and *M. floridulus* are considered bioenergy crops because of their high biomass and low nutrient requirement [18]. Both *M. sinensis* and *M. floridulus* originate from Asia and they are widely distributed across Taiwan [19]. *M. sinensis* and *M. floridulus* have distinct biogeography: In Taiwan, *M. sinensis* inhabits diverse environments with a wide range of edaphic factors, while *M. floridulus* is distributed mainly at elevations below 2000 m [19]. We used microsatellite markers to study the genetic variation of *Miscanthus*. Microsatellites are useful for characterizing the genetic structure of individual *Miscanthus* populations [20] and are ideal for surveying plant genetic diversity because of their high resolution [21].

Previous studies have reported that the host plant differentially affects bacterial and fungal communities via root-released organic carbon [22]. Additionally, the effects of host plants on microbial communities seem to depend on plant compartments [23]. Therefore, we hypothesize that the relative importance of host plant genetic variation and soil environments in influencing prokaryote vs. fungi might change from rhizosphere soil to root endophytic compartment. We also hypothesize that plant genetic variation would have a stronger effect on the core microbial community than the non-core microbial community since core microbes play more important roles in plant development. Lastly, we hypothesize that microbial co-occurrence network stability might change from rhizosphere soil to root endosphere since host plants select for the compartment-specific microbial community.

## Materials and methods

### Study design, sample collection, and soil physicochemical analysis

This study was conducted in 16 sites across Taiwan (Table S1), which were selected to represent mature native sites of *M. sinensis* and *M. floridulus* across a range of environmental factors. In Taiwan, *M. sinensis* and *M. floridulus* are mainly distributed in the north and central regions, respectively. At each sampling site, four quadrats (1 m^2^) were randomly established. For root sampling, three *Miscanthus* plants were randomly selected from each quadrat, and roots were removed with a shovel, shaken to remove loosely adhered soil, and clipped and then immediately placed in a bag. Rhizosphere soil (defined as that tightly attached to the roots) of the same plant was collected afterwards. The topsoil (0–12 cm) c. 20 cm away from the plants was collected, with six soil cores thoroughly mixed for analysis of soil chemistry for each quadrat. A total of 236 soil samples were collected from sixteen sites (see detailed sample information in Table S1). All roots, rhizosphere soils, and soil samples were transported to the laboratory on ice until further processing. Soil samples were sent to the Iowa State University Soil Test Lab (Ames, IA) for chemical analyses (e.g., pH, NH_4_^+^-N and NO_3_^−^-N, Table S2).

Rhizosphere soil was washed off *Miscanthus* root using 40 mL sterile deionized water and collected in sterile containers for characterization of rhizosphere microbial populations. Rhizosphere soil was stored at − 80 ℃ and lyophilized prior to DNA extraction. Roots were surface sterilized following the methods of Chelius and Triplett (2001) [24], with modifications. Each root was placed in a 1L container containing 100 mL 95% ethanol and shaken for 30 s. The ethanol was then replaced with 100 mL 5.25% sodium hypochlorite and shaken for 30 s. Sterilized roots were then rinsed three times with 300 mL sterile distilled water to remove all traces of sodium hypochlorite. Using ethanol-sterilized pruners, roots were chopped into small pieces (3 to 5 cm in length) and placed into a sterilized Waring blender with 30 mL phosphate buffered saline (PBS) + 0.1% Tween 80. Roots were ground in the blender and placed into sterile centrifuge tubes containing five sterile glass beads. Pulverized roots were washed gently to release endophytic microbes following the methods of Brulc et al. (2009) [25] with modifications. Root slurries were then shaken gently (at approximately 100 rpm) on ice for one hour and plant material was removed by filtration through a sterile 3-inch No. 25 US Standard Test Sieve. Endophytic prokaryote and fungi contained in the filtrate were concentrated by centrifugation prior to DNA extraction. Root endophyte extracts were stored at − 80 °C awaiting DNA extraction.

### DNA extraction, prokaryotic 16S rRNA, and fungal ITS rRNA gene amplification

Genomic DNA was extracted from 0.25 g lyophilized soil using the FastDNA Spin Kit for Soil (MP Biomedicals, Solon, OH) and from 0.25 g root material using the FastDNA Spin Kit (MP Biomedicals, Solon, OH) following the manufacturer’s protocol. DNA concentration was measured using a Qubit dsDNA HS kit (Life Technologies Inc., Gaithersburg, MD, USA). A master mix for amplification was prepared using the Roche High Fidelity Fast Start Kit and 20X Access Array loading reagent according to Fluidigm protocols. Amplicon preparation of the V4 region of the prokaryotic 16S rRNA and fungal ITS rRNA genes was carried out using a Fluidigm Access Array IFC chip (Fluidigm) with single-index barcoded primers 515F (5′-GTGYCAGCMGCCGCGGTAA-3′) and 806R (5′-GGACTACNVGGGTWTCTAAT-3) [26], and primers ITS3-F′(5′-GCATCGATGAAGAACGCAGC-3′) and ITS4-R′(5′-TCCTCCGCTTATTGATATGC-3′) [27], respectively. DNA sequencing was completed using 2 × 250 bp paired-end chemistry on a Sp flowcell in an Illumina NovaSeq 6000 Sequencing System (Illumina) at the Roy J. Carver Biotechnology Center.

### Microsatellite genotyping of *Miscanthus*

Root genomic DNA was also used for host plant genetic analysis. Sixteen previously described microsatellite loci [28, 29] allowed observations of polymorphisms in *M. sinensis* and *M. floridulus* (Table S3). DNA was amplified by PCR cycling with an initial denaturation of 5 min at 95 °C, followed by 35 cycles of 1 min at 95 °C (denaturation), 1 min at a primer-specific annealing temperature, and 1 min at 72 °C (extension), with a final extension at 72 °C for 10 min. The reaction mixture (10 µl) contained 10 × reaction buffer (New England Biolabs, Beverly, MA), 2 mM MgSO_4_, 0.125 µM dNTPs, 0.25 µM of each primer 0.5 U of Taq DNA polymerase (New England Biolabs) and 40 ng template DNA. Forward primers were then fluorescently labeled so that they could be used for automated genotyping. The PCR products were treated with poly(A) at 65 °C for 30 min, then diluted in ddH_2_O if too concentrated and sized using the LIZ500 internal sizing standard on an ABI 3130xl automated DNA sequencer with GENEMAPPER V4.0 software (Applied Biosystems, Foster City, CA).

### Host plant genetic variation

In each *Miscanthus* species, the presence and absence of the microsatellite DNA bands for each primer–individual combination was scored as either 1 or 0. Host plant population genetic distance (Nei’s genetic distance (D)) was estimated using the software of power-marker 3.25 [30]. Pairwise Nei genetic distances of host plant (pairwise sums of the branch lengths connecting terminal gene) were calculated using the cophenetic.phylo command in the Ape package [31]. Principal coordinate analysis (PCoA) was used to convert the pairwise Nei genetic distances to genetic eigenvectors using the cmdscale command in the vegan package [32]. Significant host plant genetic PCoA was forward-selected (α = 0.05) using the forward.sel command in the Packfor package [33] prior to subsequent statistical analyses.

### Bioinformatic analysis of microbes

DNA sequences were obtained as fastq files. Paired-end 16S rRNA and ITS sequences were merged using Fast Length Adjustment of SHort reads (FLASH) software [34]. Quality filtering of fastq files was performed using the FASTX-Toolkit software; primer sequences and sequence reads with a quality score of less than 30 and with fewer than 90% of bases were removed [35]. Sequences were binned into discrete operational taxonomic units (OTUs) based on 97% similarity using USEARCH [36]. Quantitative Insight into Microbial Ecology (MacQIIME version 1.9.2) was used solely for generating an OTU table and assigning taxonomy based on the Greengenes reference database for bacteria and archaea, and the UNITE database for fungi [37]. Sequences identified as plants, protists, chloroplasts, and mitochondria were removed prior to statistical analysis. A total of 59,405,332 raw reads were obtained from the V4 region. Library size ranged from 1 to 18,000 sequences per sample from the prokaryotic V4 region with a mean of 6420 sequences per sample, and 1 to 10,820 sequences per sample for the fungal ITS region with a mean of 3850 sequences per sample. Read counts were rarefied to 5970 reads for 16S rRNA rhizosphere soil and root endophytic samples, 2,157 reads for ITS rRNA rhizosphere soil and root endophytic samples. After rarefying, 185 rhizosphere soil samples and 185 root endophytic samples with 21,221 OTUs were left for 16S rRNA data; 159 rhizosphere soil samples and 159 root endophytic samples with 5391 OTUs were left for fungal ITS data. Raw sequences were submitted to Sequence Read Archive (SRA) database under accession number SUB11522211.

### Statistical analysis

### Microbial richness and community composition analysis

Most statistical analyses were conducted in R v.4.3.36. Rarefaction curves were computed for all prokaryote and fungi in rhizosphere soil and root endophyte samples collected from *M. sinensis* and *M. floridulus* to evaluate the comprehensiveness of the sampling strategy using the vegan package [38] in R. Distance matrices for the rhizosphere soil and endophytic prokaryotic and fungal community from *M. sinensis* and *M. floridulus* were constructed by calculating dissimilarity with the Bray–Curtis method on Hellinger-transformed OTU read data. To investigate patterns of rhizosphere soil and endophytic prokaryotic and fungal community structures in *M. sinensis* and *M. floridulus*, unconstrained PCoA (for principal coordinates PCo1 and PCo2) ordination of analysis was performed based on the Bray–Curtis dissimilarity matrices. Then, differences in microbial community compositions between rhizosphere soil and roots were tested by conducting a permutational multivariate analysis of variance (PERMANOVA) using the ‘adonis2’ function of vegan package in R. To control for underlying variation across sites, we restricted the permutation to be within the same site using ‘adonis2’. To further confirm the differences of microbial community compositions between rhizosphere soil and root endophyte, we also performed a partial canonical analysis of principal coordinates (CAP) to partial out site effect based on Bray–Curtis distance using “capscale” function of vegan package in R. All CAP models were tested for significance using PERMANOVA (“permutest”, permutations = 9999, *p* ≤ 0.05).

### Enriched microbial OTUs in root and rhizosphere soil of *Miscanthus* species

To identify prokaryotic and fungal OTUs enriched within the root endophyte relative to the rhizosphere soil in *M. sinensis* and *M. floridulus*, we used a negative binomial model in the R package DESeq2 [39] to model operational taxonomic unit (OTU)-level root endophyte and rhizosphere soil community abundances. OTUs were considered enriched if they had a log2-fold change greater than 2 and an adjusted *P*-value less than 0.05.

### Microbiome assembly from rhizosphere soil to root endophyte

Normalized stochasticity ratio (NST) was used to quantify the ecological stochasticity of rhizosphere soil and root endophyte in microbial communities [40]. This index measures the relative importance of stochasticity vs. determinism considering both the situations where deterministic factors drive the communities to be more similar or dissimilar than expected from random patterns [40]. An NST < 0.5 indicates the more deterministic and > 0.5 more stochastic community assembly. NST was calculated based on Jaccard similarity metrics using null model algorithm PF (fixed data richness and proportional taxa occurrence frequency). NST analysis was performed in R using the NST package [40]. In order to further confirm the NST result, the Sloan neutral model [41] was applied to the 16S rRNA and ITS rRNA community data. Fitting of the neutral model was performed in R according to Burns et al. [42]. We used a neutral community model (NCM) to predict the relationship between OTU detection frequency and their relative abundance across rhizosphere soil and root endophytic microbial community. The model used here is an adaptation of the neutral theory adjusted to large microbial populations. In general, the model predicts that taxa that are abundant in the metacommunity will be widespread, since they are more likely to disperse by chance among different sampling sites, whereas rare taxa are more likely to be lost in different sites due to ecological drift (i.e., the stochastic loss and replacement of individuals). The parameter R^2^ represents the overall fit to the neutral model [42], and a higher *R*^2^ value indicates the greater importance of stochastic processes. Calculation of 95% confidence intervals around all fitting statistics was done by bootstrapping with 1000 bootstrap replicates.

### Effects of host plant genetic variation and environment on microbial community composition

In order to study the relative contribution of host plant genetic variation and environmental factors on microbial community compositions, we conducted PERMANOVA based on Bray–Curtis dissimilarity distances to calculate the contribution of host plant (host genetic variation), soil, and site (altitude, latitude, and longitude) on microbial community compositions using the ‘adonis2’ function of the vegan package in R. To control for the underlying variation across sites, we also restricted the permutations in PERMANOVA to be within the same site using ‘adonis2’.

### Core microbiome taxa and the effect of host genetic variation on core microbial community composition

To infer the core rhizosphere and endophyte taxa and prioritize them for further inquiry, we calculated the abundance-occupancy distributions of taxa, as established in macroecology [42]. For each OTU, we calculated occupancy and mean relative abundance for each compartment in each *Miscanthus* species. Only OTUs with occupancy of 80% and relative abundance of 0.01% (found in 80% of samples for each compartment and relative abundance in 0.01% of total reads for each compartment) were prioritized as core members. Using this conservative threshold for occupancy, we included all OTUs that had strong compartment signatures; these taxa also were in high abundance and were persistent as indicated by their abundance-occupancy distributions. We quantified the explanatory value of the core members to community assembly using a previously published method of partitioning community dissimilarity:

$$C=\frac{\mathrm{BC}core}{\mathrm{BC}all}$$

where C is the relative contribution of community Bray-Curtis (BC) dissimilarity attributed to the core OTUs. In order to test the effect of host plant genetic variation, soil and site on core and non-core microbial community composition in rhizosphere soil and root in each *Miscanthus* species, we used PERMANOVA with the “adonis2” function of the vegan package in R based on Bray–Curtis dissimilarity distances. To control for underlying variation across sites, we also restricted the permutations in PERMANOVA to be within the same site using “adonis2.”

### Microbial co-occurrence network stability in rhizosphere soil and root endophyte

In order to understand how microbial interactions changed from rhizosphere soil to the root endophytic compartment in *M. sinensis* and *M. floridulus*, the prokaryotic and fungal networks were constructed using the “WGCNA” R package based on the Spearman correlation index [43]. The nodes and the edges in the network represent prokaryotic and fungal OTUs and the significant interactions between pairs of OTUs, respectively. The OTUs with relative abundances less than 0.01% were filtered because they were poorly represented [44]. The *P*-values for multiple testing were calculated using the Benjamini and Hochberg false discovery rate (FDR) test controlling procedure [45]. Only the rank correlation coefficient with values above 0.7 or below -0.7 and a statistically significant adjusted-P value lower than 0.001 were considered as a valid correlation in the network. Sub-networks for each individual sample from the meta-community network, were then identified by preserving prokaryotic and fungal OTUs present in each plant using the “igraph” package [46]. The networks of the rhizosphere soil and endophytes were graphically displayed in Gephi (http://gephi.github.io/). Erdös-Réyni model random networks with the same number of nodes and edges as the observed networks were also constructed for each compartment in each *Miscanthus* species. To quantify the response of microbial interactions from rhizosphere soil to root endosphere under host selection, we then quantified two network properties including modularity and cohesion values, which have been used to evaluate microbial community stability in previous studies [47, 48]. The Wilcoxon test was then employed to assess significant differences in measured topological parameters between endophytic and rhizosphere soil networks. A module is a group of nodes that are highly connected within the group and less connected outside the group. Modules were detected using the greedy modularity optimization method [49].

## Results

### Characterization of sequencing data

There were 1 to 163,044 and 1 to 25,669 reads per sample for 16S rRNA and ITS sequencing in root and rhizosphere soil, respectively. We rarefied samples to 5970 reads per sample for 16S rRNA gene amplicons and to 2157 for ITS. With these thresholds, we achieved richness asymptotes for both datasets, suggesting that sequencing efforts were sufficient to capture comparative dynamics and diversity (Fig. S1). The total richness observed at this rarefaction depth was 5391 fungal and 21,221 prokaryotic OTUs. The prokaryotes were dominated by Proteobacteria, Actinobacteria, and Acidobacteria in both *M. sinensis* and *M. floridulus*. The relative abundance of Proteobacteria was higher in root endophyte than in rhizosphere soil, whereas Actinobacteria and Acidobacteria showed higher relative abundance in rhizosphere soil than in root endophyte communities for both in *Miscanthus* species (Fig. S2A; Fig. S3A). The fungi were dominated by Ascomycota and Basidiomycota both in *M. sinensis* and *M. floridulus*, with varying relative abundances in root endophyte assemblages and rhizosphere soil (Fig. S2B; Fig. S3B).

### Microbiome assembly from rhizosphere soil to root endophyte

**Fig. 1:**
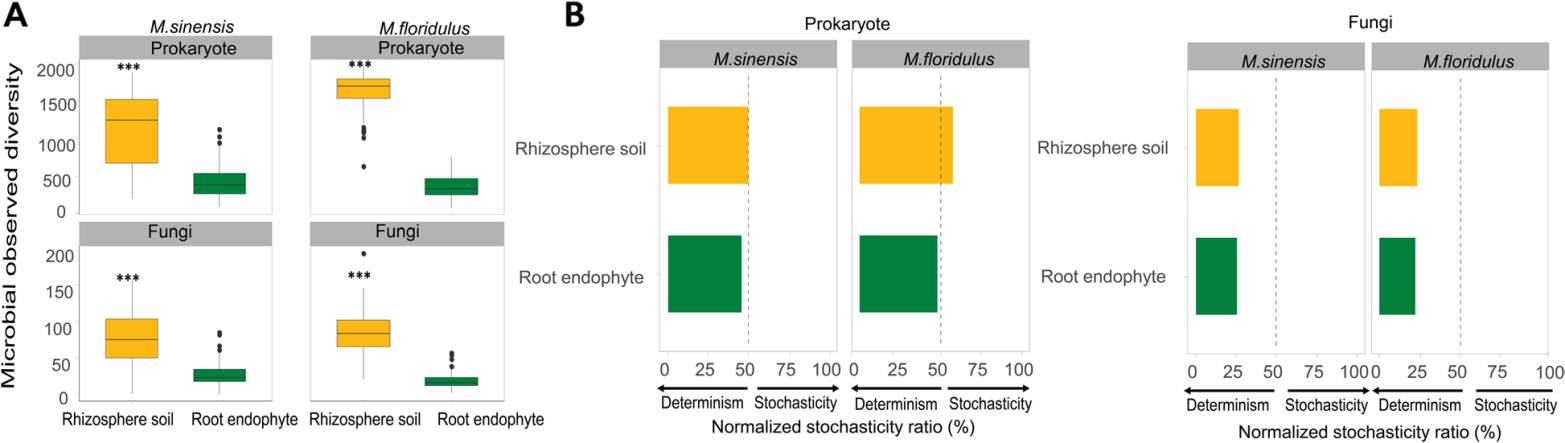
The diversity and stochasticity of prokaryotic and fungal communities in rhizosphere soil and root endophyte of *M. sinensis* and *M. floridulus*. **A** OTU richness. Horizontal lines within boxes denote medians. Top and bottom segments of the boxes denote the 75th and 25th percentiles, respectively. Upper and lower whiskers extend to data no more than 1.59 the interquartile range from the upper edge and lower edge of the box, respectively. Asterisks indicate significant differences among plant microhabitats (*P* < 0.001), based on Kruskal–Wallis one-way test. **B** The normalized stochasticity ratio (NST) of prokaryotic and fungal communities under different plant compartments in *M. sinensis* and *M. floridulus* developed based on Jaccard distances with 50% as the boundary point between more deterministic (< 50%) and more stochastic (> 50%) community assembly

Total prokaryotic and fungal OTU richness was significantly higher in the rhizosphere soil than in root endophyte in both *M. sinensis* and *M. floridulus* (Fig. 1A; Fig. S4A, B). The PCoA ordinations analysis showed that the prokaryotic and fungal community assemblies were clearly distinct between rhizosphere soil and root (Fig. S5). Furthermore, after accounting for site effects, we still found significant differences in the microbial community compositions between root endosphere and rhizosphere soil both in *M. sinensis* and *M. floridulus* (PERMANOVA: *p* < 0.0001 for both prokaryotes and fungi). The CAP ordinations also showed the divergence between rhizosphere soil and root endophytic communities while controlling for site effect (Fig. S6). Taken together, PCoA, partial CAP controlling for site effect, and PERMANOVA permutated within the site all confirmed the significant differences in the microbial community compositions between root endophytes and rhizosphere soil in both miscanthus species.

The NST ratio was calculated based on OTU matrices (Bray–Curtis). Results indicated that the prokaryotic and fungal communities were more strongly driven by deterministic assembly processes (NST < 50%), with root endophyte communities exhibiting a higher deterministic ratio than rhizosphere soil microbiomes (Fig. 1B). The Sloan neutral model was well fitted to the prokaryotic and fungal communities for all plant compartments, with a lower *R*^2^ value in root endophytic than in rhizosphere soil communities (Fig. S7). We also found NST ratio and Sloan neutral model R^2^ values of fungal communities were lower than those of prokaryotic communities in both *Miscanthus* species, suggesting fungal communities were more strongly driven by deterministic assembly processes (Fig. 1B). Together with the neutral community model, we confirmed that deterministic processes became more important in shaping microbiome assembly in the transition from rhizosphere soil to root endosphere for both *Miscanthus* species.

### Enriched microbial OTUs in root and rhizosphere soil of *Miscanthus* species

**Fig. 2:**
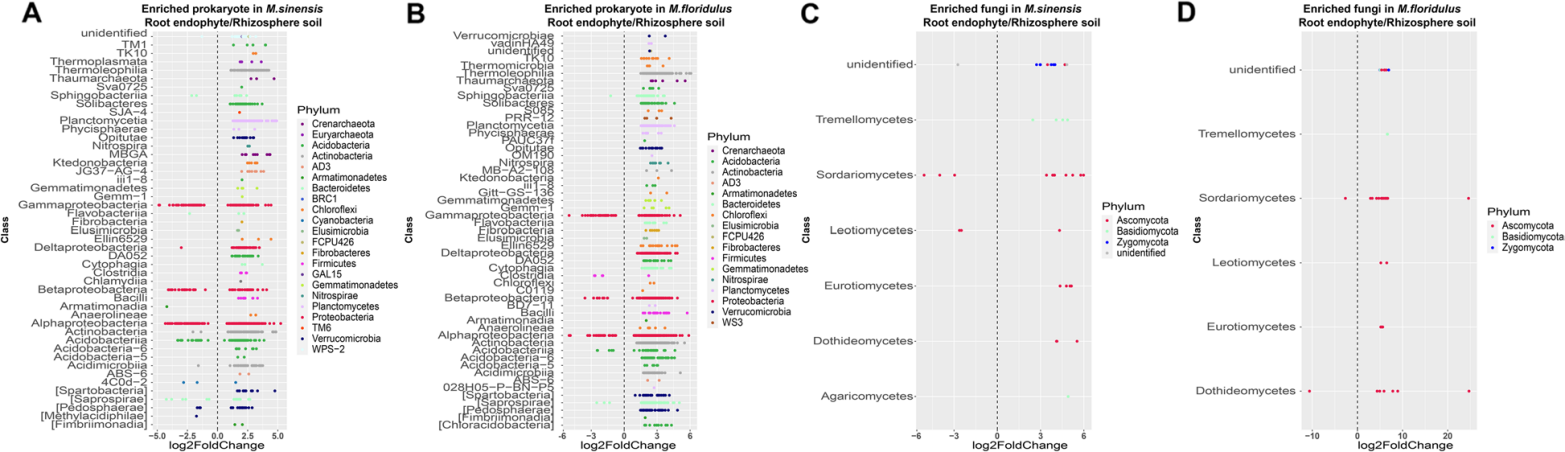
Enriched microbial OTUs inhabiting different plant root microhabitats. Enrichment (positive) and depletion (negative) of OTUs between root endophyte and rhizosphere soil in *M. sinensis* prokaryotic community (**A**), *M. floridulus* prokaryotic community (**B**), *M. sinensis* fungal community (**C**), and *M. floridulus* fungal community (**D**). Each point represents an individual OTU

The enrichment of specific prokaryotic and fungal OTUs in the root endophyte and rhizosphere soil in each *Miscanthus* species was identified using DESeq2 (Fig. 2). Our results showed that the numbers of rhizosphere-enriched microbial OTUs were higher than that of root-enriched microbial OTUs in the two *Miscanthus* species. We also found that root-enriched prokaryotic OTUs belonged to Gammaproteobacteria, Alphaproteobacteria, Betaproteobacteria, Sphingobacteriia and [Saprospirae] both in *M. sinensis* and *M. floridulus*; while enriched prokaryotic taxa in the rhizosphere were widely distributed among different phyla (Fig. 2A, B). Fungal OTUs included Sordariomycetes and Leotiomycetes that were enriched in roots of *M. sinensis* and Sordariomycetes and Dothdieomycetes that were enriched in roots of *M. floridulus*; fungal taxa enriched in rhizosphere soil were widely distributed among different phyla (Fig. 2C, D, Table S4).

### Effects of host plant genetic variation and environment on microbial community composition

**Fig. 3:**
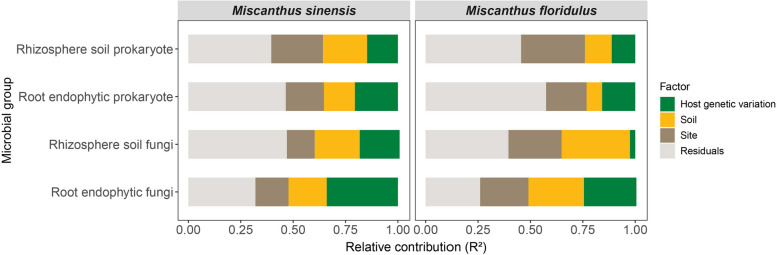
Relative contribution of the different predictors used to model prokaryotic and fungal community compositions in rhizosphere soil and root endophyte in *M. sinensis* and *M. floridulus*. Panels represent results from PERMANOVA aiming to identify the percentage of variance of rhizosphere soil and root endophyte prokaryotic and fungal community compositions of explained by host genetic variation, soil variables, and site in *M. sinensis* and *M. floridulus*. An alternative version of this figure showing each group of predictors can be found in Table S5

Our results based on PERMANOVA analysis suggested that the variations in root endophytic and rhizosphere soil prokaryotic and fungal communities of *Miscanthus* were mainly explained by site, host genetic variation, and soil factors (Fig. 3, Table S5). The importance of host genetic variation on prokaryotic and fungal community composition increased from rhizosphere soil to root endophytic communities in both *Miscanthus* species (Fig. 3, Table S5). Furthermore, we also found host plant genetic variation has a greater impact on fungal community composition than prokaryotic community composition in rhizosphere soil and root endophyte for both *Miscanthus* species (Fig. 3).

### Core microbiome and the effect of host plant genetic variation on core microbial community compositions

**Fig. 4:**
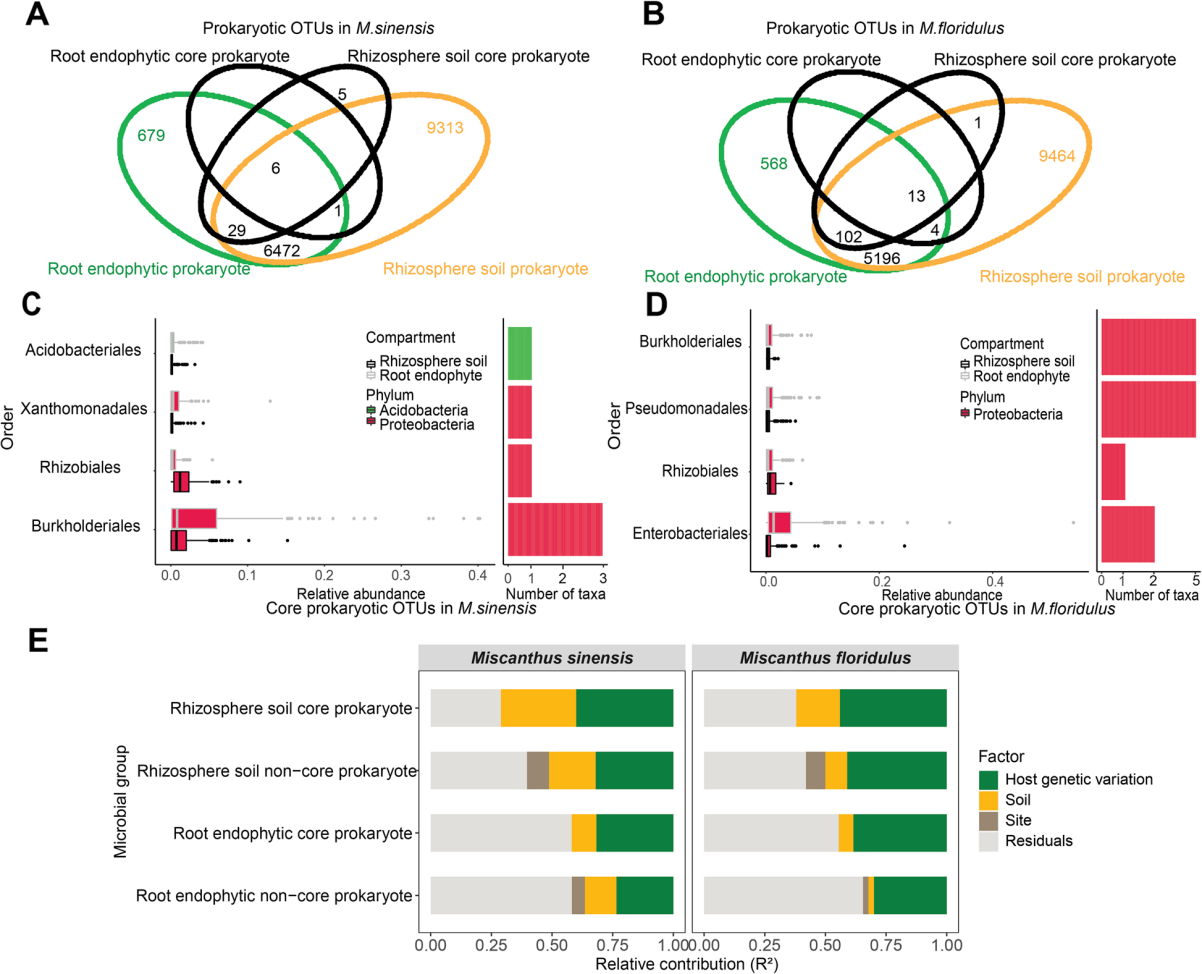
Core prokaryotes and the relative contribution of the different predictors used to model core and non-core prokaryotic community compositions in rhizosphere soil and root endophyte in *M. sinensis* and *M. floridulus*. Venn diagram showed that there are 6 and 13 core prokaryotic taxa shared between root and rhizosphere soil in *M. sinensis* (**A**) and *M. floridulus* (**B**), respectively; **C** Relative abundance of the 6 rhizosphere-root shared core taxa is represented as boxplots (left panel), grouped by order and dataset (rhizosphere soil /root) in *M. sinensis*; **D** Relative abundance of the 13 rhizosphere-root shared core taxa is represented as boxplots (left panel), grouped by order and dataset (rhizosphere soil /root) in *M. floridulus*; **E** Panels represent results from PERMANOVA aiming to identify the percentage of variance of rhizosphere soil and root endophyte core prokaryotic and non-core prokaryotic community compositions of explained by host genetic variation, soil variables, and site in *M. sinensis* and *M. floridulus*. An alternative version of this figure showing each group of predictors can be found in Table S6

To explore the core microbiome from rhizosphere soil and the root endophytic compartment in two *Miscanthus* species, we calculated the abundance-occupancy distributions of prokaryotic and fungal OTUs (Fig. S8). We found the number of core prokaryotic OTUs varied between root endophyte and rhizosphere soil and between two *Miscanthus* species (Fig. 4A, B). The core prokaryotic OTUs consisted of c. 0.09–0.726% of total prokaryotic OTUs (Fig. 4A, B), yet represented 13.7–22.7% of the sequences. The core prokaryotic OTUs were not closely associated with the overall prokaryotes, as reflected by the low contribution of core prokaryotic community variation to the total prokaryotic community variation in both *Miscanthus* species (Fig. S8 B, D, F, H). The order level of taxonomy for core prokaryotic OTUs in each plant compartment can be seen in Fig. S8 (A, C, E, F). The shared core prokaryotic OTUs between rhizosphere soil and root were mainly members of Proteobacteria and Acidobacteria in both *Miscanthus* species (Fig. 4C, D). However, no core fungal OTUs were identified in these *Miscanthus* species.

We also tested how host genetic variation affects core and non-core prokaryotic community composition in roots and rhizosphere soil of both *Miscanthus* species. Our results based on PERMANOVA analysis showed that host genetic variation explained most of the microbial community variations in root and rhizosphere soil for both *Miscanthus* species (Fig. 4E, Table S6). Additionally, the effect of host genetic variation on core prokaryotic community composition was greater than that of non-core prokaryotic community composition for both *Miscanthus* species (Fig. 4E, Table S6).

### Microbial co-occurrence network stability in rhizosphere soil and root endophyte

**Fig. 5:**
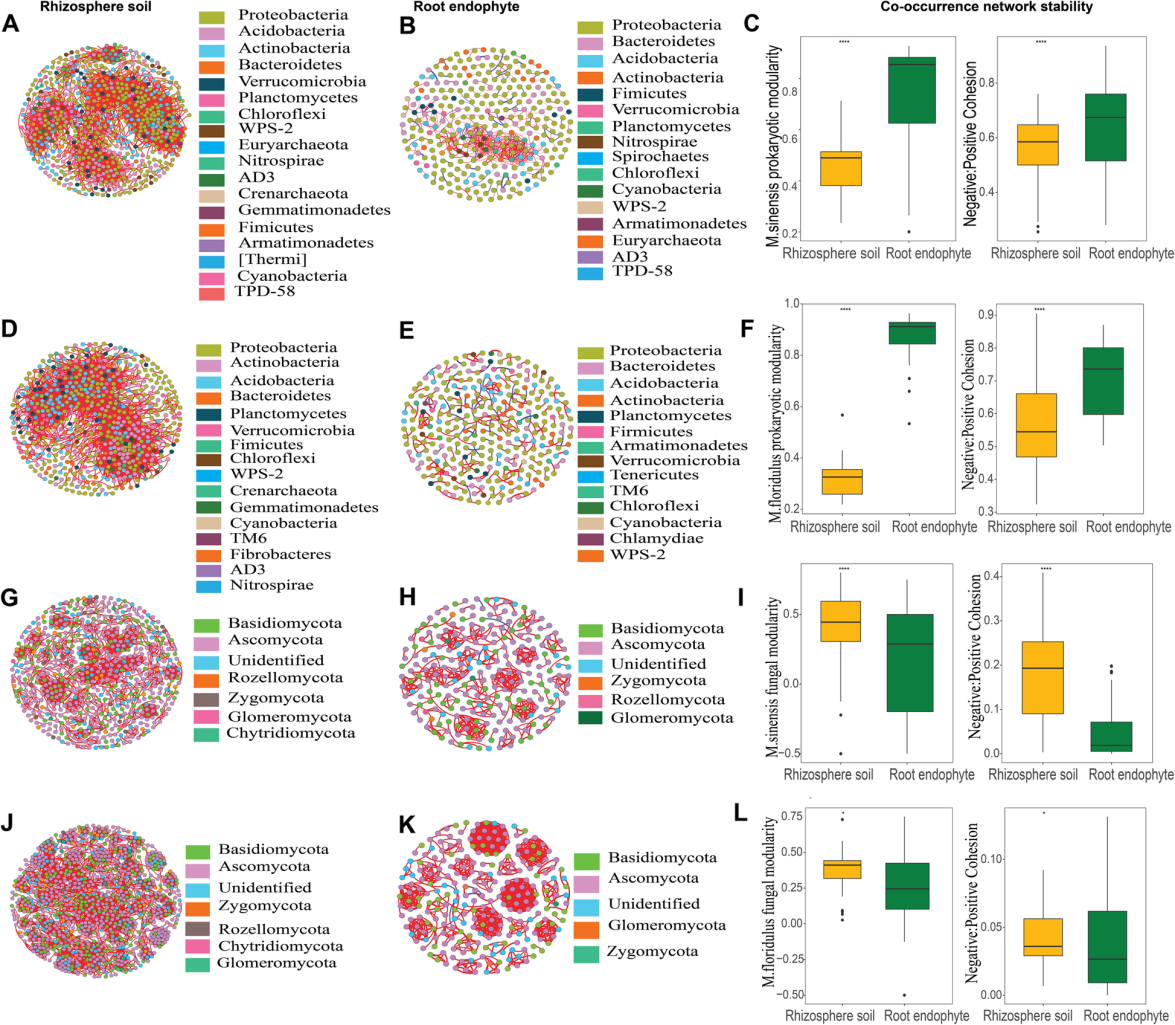
Prokaryotic and fungal co-occurrence network patterns in rhizosphere soil and root endophyte in *M. sinensis* and *M. floridulus*. Panels **A**, **D**, **G**, and **J** show the rhizosphere soil prokaryotic co-occurrence network in *M. sinensis*, rhizosphere soil prokaryotic co-occurrence network in *M. floridulus*, rhizosphere soil fungal co-occurrence network in *M. sinensis*, rhizosphere soil fungal co-occurrence network in *M. floridulus*; panels **B**, **E**, **H**, and **K** show the root endophytic prokaryotic co-occurrence networks in *M. sinensis*, root endophytic prokaryotic co-occurrence network in *M. floridulus*, root endophytic fungal co-occurrence network in *M. sinensis*, root endophytic fungal co-occurrence network in *M. floridulus*. Panels **C**, **F**, **I**, and **L** show differences in co-occurrence topological features specifically modularity and negative: positive cohesion ratio between rhizosphere soil and root endophyte in *M. sinensis* and *M. floridulus*. Asterisks indicate the significant values in compartment-enriched taxa (**p < 0.01; ***p < 0.001; Wilcoxon rank-sum test)

To quantify the changes of microbial interactions from rhizosphere soil to roots, we assessed the co-occurrence patterns of prokaryotic and fungal communities in both *Miscanthus* species (Fig. 5). The prokaryotic and fungal co-occurrence networks in rhizosphere soil differed profoundly from the endophytic networks in root for both *Miscanthus* species (Fig. 5, Table S7). Furthermore, root endophytic prokaryotic communities had higher network stability than fungi as evidenced by higher modularity and cohesion (Fig. 5C, F). Prokaryotic community networks increased in the modularity and the ratio of negative to positive cohesion from rhizosphere soil to root both in *M. sinensis* and *M. floridulus* (Fig. 5C, F), but fungal community network decreased in modularity and the ratio of negative to positive cohesion from rhizosphere soil to root (Fig. 5I, L).

## Discussion

### Enriched microbial OTUs and core microbial taxa in *Miscanthus* species

We found that members within Gammaproteobacteria, Alphaproteobacteria, Betaproteobacteria, Sphingobacteriia, and [Saprospirae] were significantly enriched in the root for both *Miscanthus* species, whereas a broad range of taxa were enriched in the rhizosphere soil. The enriched endophytic bacteria identified in miscanthus in this study have also been detected in other plants such as rice, barley, and *Arabidopsis thaliana* [50–52]. These root-enriched microbial taxa may play key roles in modulating host nitrogen uptake and host fitness [53]. For example, we found members of enriched taxa belonged to the genera of *Azotobacter*, *Azospirillum*, *Enterobacter*, *Herbaspirillum*, and *Rhizobium* that are known diazotrophs [54], which could have contributed to the nitrogen fixation in the *M. sinensis* and *M. floridulus* root. Future research is needed to understand the contribution of N fixation to the N budget of miscanthus, and the ability to recruit endophytic diazotrophs would do much to promote the sustainability of this candidate bioenergy crop [55, 56]. The higher number of prokaryotic phyla enriched in the rhizosphere soil for both *Miscanthus* species suggests that rhizosphere soil may have greater functional diversity and/or redundancy for biogeochemical cycling functions compared with root endophytic communities.

We also identified a group of *Miscanthus* core prokaryotic taxa shared between the rhizosphere soil and the root (Fig. 4A–D). Most members of the *Miscanthus* core prokaryotes (e.g. Acidobacteriales, Xanthomonadales, Rhizobiales, Burkholderiales, and Enterobacteriales) overlapped with those identified in other plant species such as *Arabidopsis thaliana* [51, 57] and sugarcane [58], suggesting that the presence of some core microbial taxa may be common across plant species. Multiple members affiliated with these core species have been verified to exert different types of positive functions on plant health and growth [13]. The existence of common core microbiota members in various host plants implies that a highly conserved, coevolutionary, and host-independent core plant microbiota may exist that maintains plant holobiont fitness [53, 57, 59].

### Host plant genetic variation differentially affected root endophytic and rhizosphere soil microbial community composition

We found that the influence of host genetic variation on microbial community composition increased from rhizosphere soil to root endophyte, which is consistent with a study on *M. truncatula* [60]. This might be due to an increase in host plant selection for microbial communities in the root compartment relative to the rhizosphere soil [52, 61]. We also demonstrated that host genetic variation imposed stronger selection on the fungal community than on the prokaryotic community for both *Miscanthus* species (Fig. 3). The different effects of host genetic variation on fungal and prokaryotic community compositions may be explained by close fungal associations with plants (compared to prokaryotes) since some fungi can form biotrophic interactions with plants, and take the form of root symbionts, endophytes, and pathogens [62].

### Host genetic variation differentially affected core and non-core prokaryotic community composition

We demonstrated that host genetic variation has a stronger effect on shaping core prokaryotic community assemblages than non-core prokaryotic community composition in roots and rhizosphere soil of the two *Miscanthus* species (Fig. 4E). Potential explanations include core microbiota traits for efficient colonization, nutrient acquisition, and stress tolerance [58, 63], which are likely to be particularly important for the host fitness and could result in more close associations with host plants. Although we found that host genetic variation was strongly related to the core prokaryotic community variation in *Miscanthus*, a previous study on common bean demonstrated no correlation between plant genotypes and core bacterial community [16]. This difference may be because our study employed a microsatellite approach, which is ideal for characterizing plant genetic variation at individual or population level. In comparison, sampling soil simply from different plant genotypes might suffer from inseparable effects between plant genetics and soil environments.

We did not observe core fungal taxa in root or rhizosphere soil in either *M. sinensis* or *M. floridulus*. This could be explained by the fact that fungi possess traits with a higher degree of resource specialization and host plant specification compared with prokaryotes [64]. In addition, fungi have relatively lower dispersal ability compared with prokaryotes [65], which leads to difficulty in the identification of common core fungal taxa among broader geographic sites as in this study.

### Microbial co-occurrence network stability changed from rhizosphere soil to root endophyte

We found prokaryotic networks in the root compartment were highly modular and dominated by negative interactions compared to rhizosphere soil. On the contrary, fungal network in root compartment had low modularity and was dominated by positive interactions compared to rhizosphere soil (Fig. 5). An increase in network modularity and the ratio of negative to positive cohesion from rhizosphere soil to root endosphere support the evidence that the host stabilizes prokaryotic communities by restricting species’ responses within small network modules, thereby avoiding propagation of the effect to the remaining network [66, 67]. The differences in the co-occurrence patterns between prokaryotes and fungi suggest that hosts might face contrasting tradeoffs between network stability and metabolic efficiency based on Coyte et al. (2015) [66]: for prokaryotic communities, we observed increased negative interactions in the host-associated microbial communities that could improve ecological stability, but at the cost of decreasing overall metabolic efficiency. However, for fungal communities, hosts might benefit from fungal cooperation to improve metabolic efficiency such that host plants prioritize the positivity of network interactions over ecological stability.

## Conclusions

In this study, we provide comprehensive and empirical evidence on the relative contribution of host and environmental factors to microbiome assembly in *M. sinensis* and *M. floridulus*. Our results demonstrate that microbiome assembly is shaped predominantly by host genetic variation, environmental factors, and biogeography. Furthermore, we revealed that host selection reduced root microbial diversity and network complexity compared to the rhizosphere soil. In addition, we also demonstrated that host genetic variation influenced fungal communities more than prokaryotic communities in both roots and rhizosphere soil. These findings significantly advance our current understanding of microbial community assembly in bioenergy crops such as *Miscanthus* under different environmental selection pressures and highlight the importance of the host selection effect for endophytic functions. Moreover, we provide empirical evidence of ecological filtering from rhizosphere soil to root endophyte compartment and selective enrichment of specific microbial taxa. *Miscanthus* root appears to select some taxa related to nitrogen fixation, which might contribute to native *Miscanthus* plant fitness and adaptation to diverse environmental conditions and signal desirable sustainability traits for *Miscanthus* as a bioenergy feedstock. We further revealed that the variation in the core microbial community was highly associated with host genetic variation in each *Miscanthus* species. The results of this study have implications for future bioenergy crop management by providing baseline data to inform translational research to harness the plant microbiome to sustainably increase agriculture productivity.

### Supplementary Information

**Additional file 1:** **Table S1.** Site name, locations, plant species of Miscanthus collecting sites. **Table S2.** The edaphic values of the sampling sites. **Table S3.** Microsatellite SNP information. **Table S4.** List of enriched fungal OTUs in different compartments, their taxonomic information, and relative abundances. **Table S5.** Results of endophyte and rhizosphere prokaryotic and fungal community compositions predicted by significantly environmental variables by PERMANOVA models. **Table S6.** Results of endophyte and rhizosphere core and non-core prokaryotic community compositions predicted by significantly environmental variables by PERMANOVA models. **Table S7.** Topological properties of microbial co-occurrence networks and their associated random networks in each compartment in *Miscanthus sinensis* and *Miscanthus floridulus*.

**Additional file 2:** **Figure S1.** Rarefaction curves for 16S rRNA of *M. sinensis* (A) and *M. floridulus* (B), and ITS rRNA of *M. sinensis* (C) and *M. floridulus* (D) dataset. **Figure S2.** Taxonomic composition of the prokaryotic (A) and fungal communities (B) in rhizosphere soil and root endophytic at the phylum level. **Figure S3.** Taxonomic composition of the prokaryotic (A) and fungal communities (B) in rhizosphere soil and root endophytic datasets of *M. sinensis* and *M. floridulus* at the phylum level. **Figure S4.** The OTU richness  of prokaryotes (A) and fungi (B) in rhizosphere soil and root endophytic datasets of *M. sinensis* and M. floridulus. **Figure S5.** PCoA plot depicting the composition patterns of prokaryotic and fungal communities from rhizosphere soil to root endophyte based on Bray–Curtis distances. **Figure S6.** Partial Canonical analysis of Principal Coordinates (CAP) of rhizosphere soil and root endophytic prokaryotic and fungal communities in *M. sinensis* and *M. floridulus*. **Figure S7.** Fit of the neutral community model (NCM) of community assembly. **Figure S8.** Core prokaryote in the rhizosphere soil and root endophyte of *M. sinensis* and *M. floridulus*.

## Data Availability

All raw sequencing data have been submitted to the NCBI Sequence Read Archive (SRA) database under accession number SUB11522211.
