# Supplementary material for: Host genetic variation drives the differentiation in the ecological role of the native Miscanthus root-associated microbiome
Source: Microbiome. 2023 Sep 30;11:216. doi: 10.1186/s40168-023-01646-3 (PMC10541700; doi:10.1186/s40168-023-01646-3)
Supplement: Supplementary file 3 — Additional file 2: Figure S1. Rarefaction curves for 16S rRNA of M. sinensis (A) and M. floridulus (B), and ITS rRNA of M. sinensis (C) and M. floridulus (D) dataset. Figure S2. Taxonomic composition of the prokaryotic (A) and fungal communities (B) in rhizosphere soil and root endophytic at the phylum level. Figure S3. Taxonomic composition of the prokaryotic (A) and fungal communities (B) in rhizosphere soil and root endophytic datasets of M. sinensis and M. floridulus at the phylum level. Figure S4. The OTU richness of prokaryotes (A) and fungi (B) in rhizosphere soil and root endophytic datasets of M. sinensis and M. floridulus. Figure S5. PCoA plot depicting the composition patterns of prokaryotic and fungal communities from rhizosphere soil to root endophyte based on Bray–Curtis distances. Figure S6. Partial Canonical analysis of Principal Coordinates (CAP) of rhizosphere soil and root endophytic prokaryotic and fungal communities in M. sinensis and M. floridulus. Figure S7. Fit of the neutral community model (NCM) of community assembly. Figure S8. Core prokaryote in the rhizosphere soil and root endophyte of M. sinensis and M. floridulus. [file 40168_2023_1646_MOESM2_ESM.docx]

**Supplementary material**

**Host genetic variation drives the differentiation in the ecological role of the native *Miscanthus* root-associated microbiome**

Niuniu Ji^1,2*^, Di Liang^1,2^, Lindsay V. Clark^3^, Erik J. Sacks^1,3^, Angela D. Kent^1,2,4*^

^1^DOE Center for Advanced Bioenergy and Bioproducts Innovation, University of Illinois at Urbana-Champaign, Urbana, IL, 61801, USA

^2^Institute for Sustainability, Energy and Environment, University of Illinois at Urbana-Champaign, Urbana, IL, 61801, USA

^3^Department of Crop Sciences, University of Illinois at Urbana-Champaign, Urbana, IL, 61801, USA

^4^Department of Natural Resources and Environmental Sciences, University of Illinois at Urbana-Champaign, Urbana, IL, 61801, USA

**Email address:**

Niuniu Ji: niuniuji@illinois.edu

Di Liang: diliang2@illinois.edu

Lindsay V. Clark: Lindsay.Clark@seattlechildrens.org

Erik J. Sacks: esacks@illinois.edu

Angela D Kent: akent@illinois.edu

**Author for correspondence:**

Niuniu Ji

Email: niuniuji@illinois.edu

Angela D. Kent

Email: akent@illinois.edu

**Contents**

**Supplementary Figures 1-8**

**Supplementary Tables 1-7**

**Supporting Figure legends**

**Figure S1. Rarefaction curves for 16S rRNA of *M. sinensis* (A) and *M. floridulus* (B), and ITS rRNA of *M. sinensis* (C) and *M. floridulus* (D) dataset.**

**Figure S2. Taxonomic composition of the prokaryotic (A) and fungal communities (B) in rhizosphere soil and root endophyte at the phylum level.**

**Figure S3. Taxonomic composition of the prokaryotic (A) and fungal communities (B) in rhizosphere soil and root endophytic datasets of *M. sinensis* and *M. floridulus* at the phylum level.**

**Figure S4. The OTU richness of prokaryotes (A) and fungi (B) in rhizosphere soil and root endophytic datasets of *M. sinensis* and *M. floridulus*.**

**Figure S5. PCoA plot depicting the composition patterns of prokaryotic and fungal communities from rhizosphere soil to root endophyte based on Bray–Curtis distances.**

**Figure S6. Partial Canonical analysis of Principal Coordinates (CAP) of rhizosphere soil and root endophytic prokaryotic and fungal communities in *M. sinensis* and *M. floridulus*.**

**Figure S7. Fit of the neutral community model (NCM) of community assembly.**

**Figure S8. Core prokaryote in the rhizosphere soil and root endophyte of *M. sinensis* and *M. floridulus*.**

**Supplementary Tables (All of these Supplementary Tables are summarized in an Excel file.)**

**Table S1**. Site name, locations, plant species of Miscanthus collecting sites.

**Table S2.** The edaphic values of the sampling sites.

**Table S3.** Microsatellite SNP information.

**Table S4.** List of enriched fungal OTUs in different compartments, their taxonomic information, and relative abundances.

**Table S5.** Results of endophyte and rhizosphere prokaryotic and fungal community compositions predicted by significantly environmental variables by PERMANOVA models.

**Table S6.** Results of endophyte and rhizosphere core prokaryotic and non-core prokaryotic community compositions predicted by significantly environmental variables by PERMANOVA models.

**Table S7.** Topological properties of microbial co-occurrence networks and their associated random networks in each compartment in *Miscanthus sinensis* and *Miscanthus floridulus*.

**Figure S1**


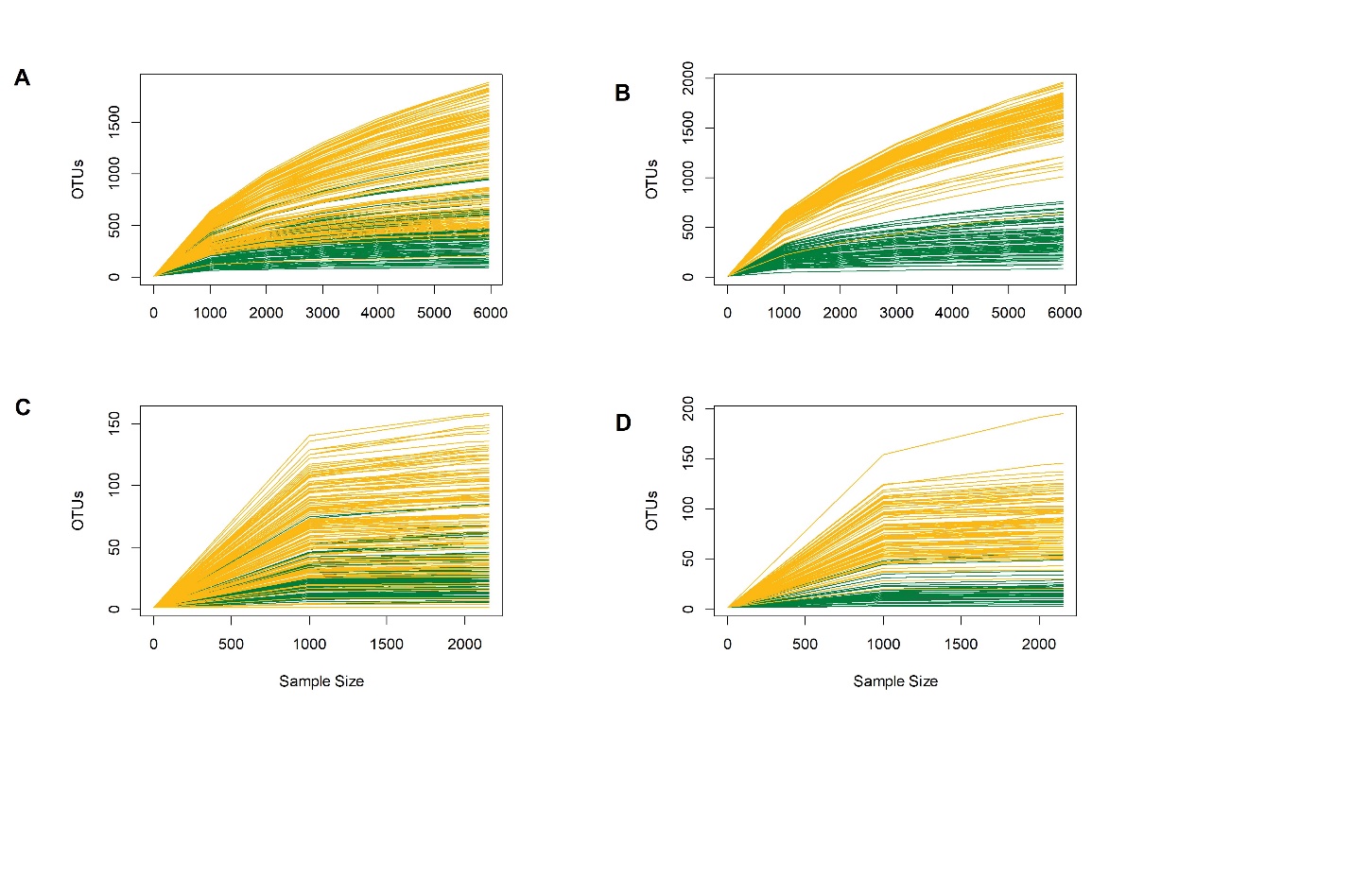


**Figure S1. Rarefaction curves for 16S rRNA of *M. sinensis* (A) and *M. floridulus* (B), and ITS rRNA of *M. sinensis* (C) and *M. floridulus* (D) dataset.** Green lines represent root endophytic samples, and the yellow lines represent rhizosphere soil samples.

**Figure S2**


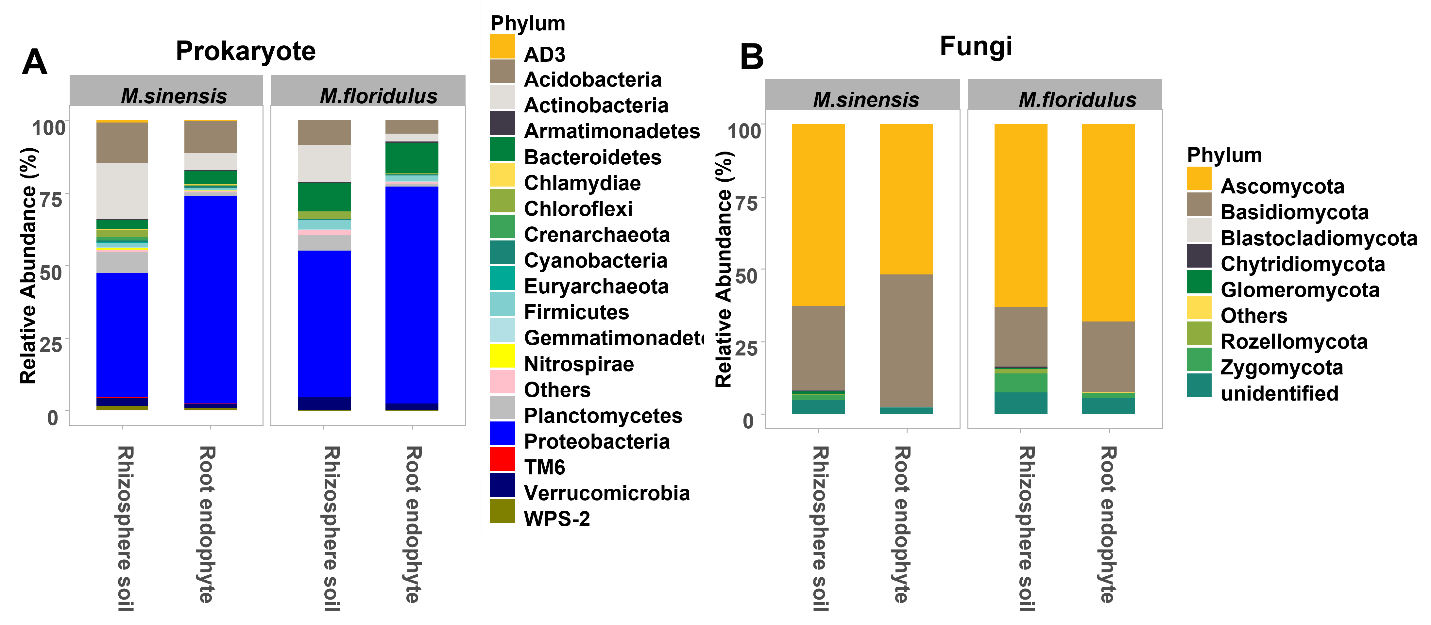


**Figure S2. Taxonomic composition of the prokaryotic (A) and fungal communities (B) in rhizosphere soil and root endophytic at the phylum level.**

**Figure S3**


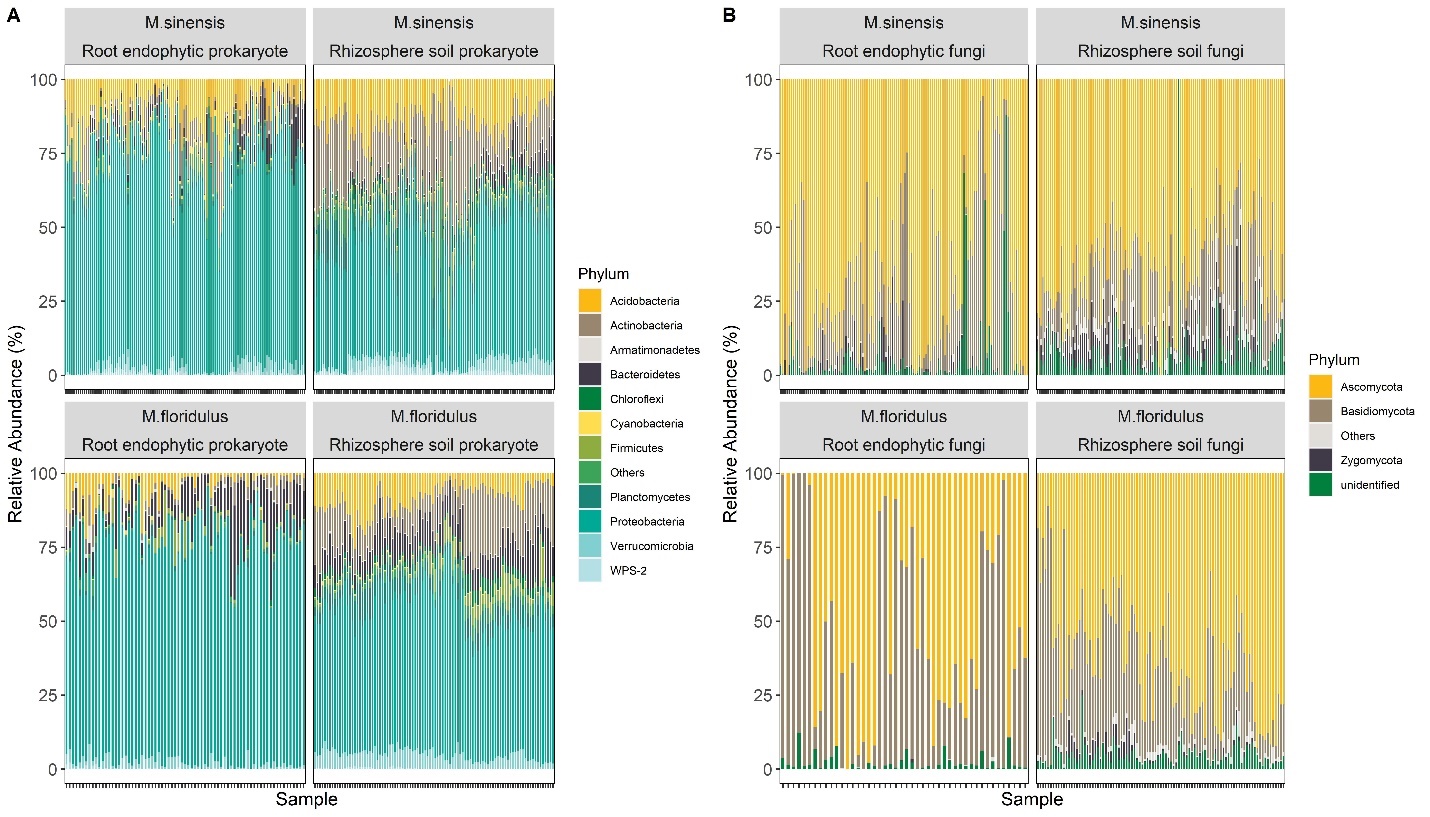


**Figure S3. Taxonomic composition of the prokaryotic (A) and fungal communities(B) in rhizosphere soil and root endophytic datasets of *M. sinensis* and *M. floridulus* at the phylum level.**

**Figure S4**


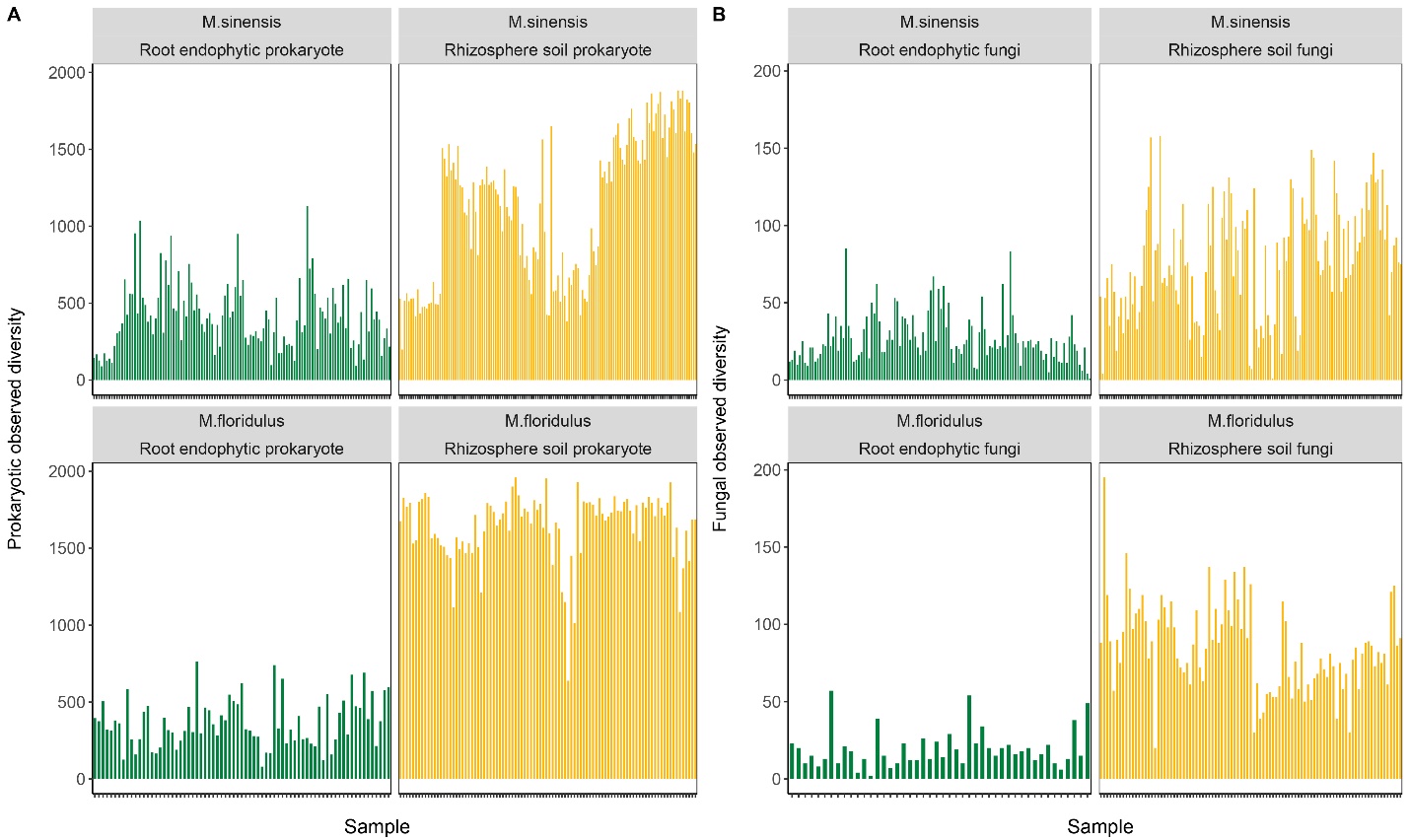


**Figure S4. The OTU richness of prokaryotes (A) and fungi (B) in rhizosphere soil and root endophytic datasets of *M. sinensis* and *M. floridulus***

**Figure S5**


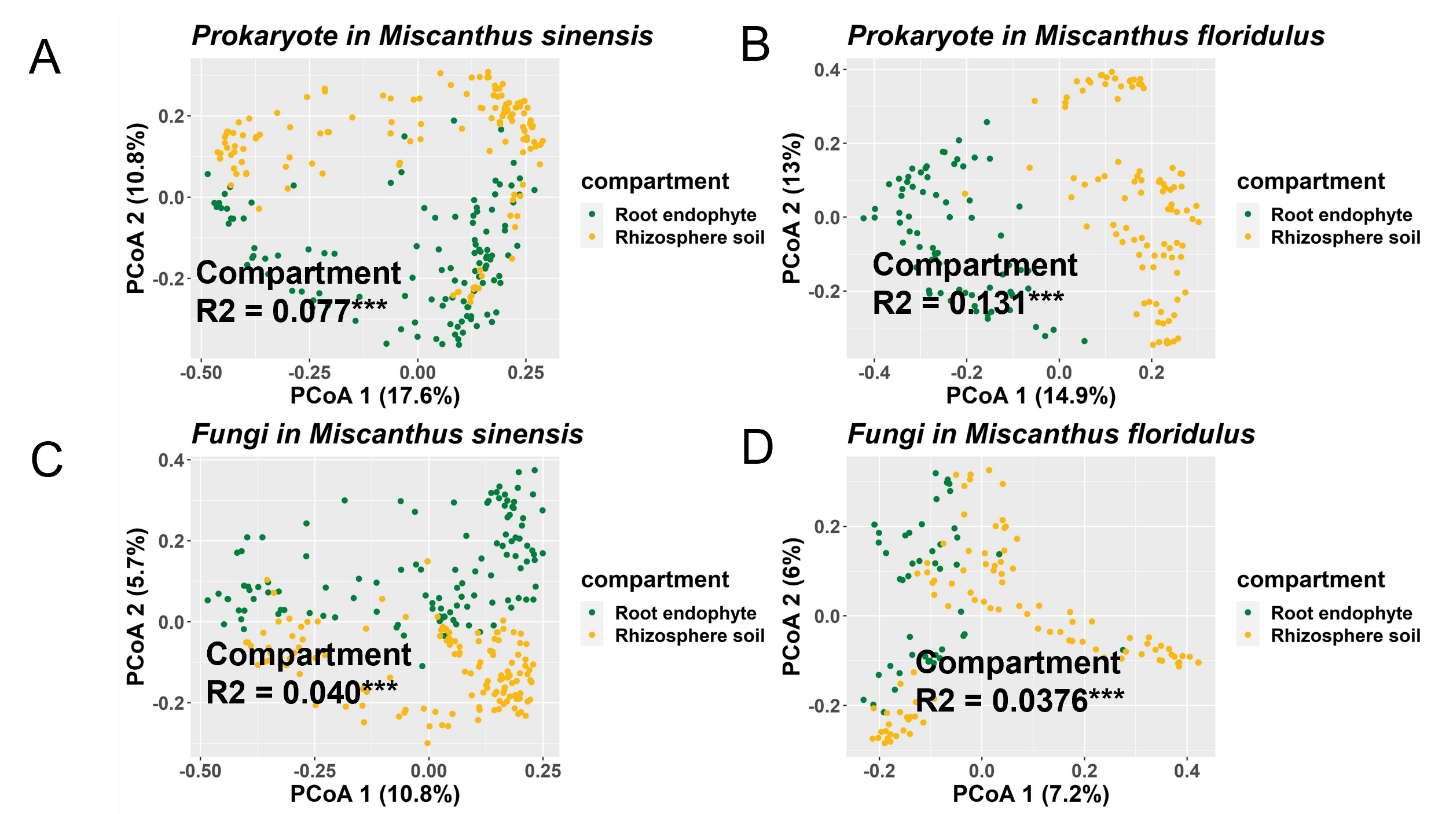


**Figure S5. PCoA plot depicting the composition patterns of prokaryotic and fungal communities from rhizosphere soil to root endophyte based on Bray–Curtis distances.** Significance of prokaryotic and fungal community dissimilarities among different plant microhabitats are based on PERMANOVA tests.

**Figure S6**


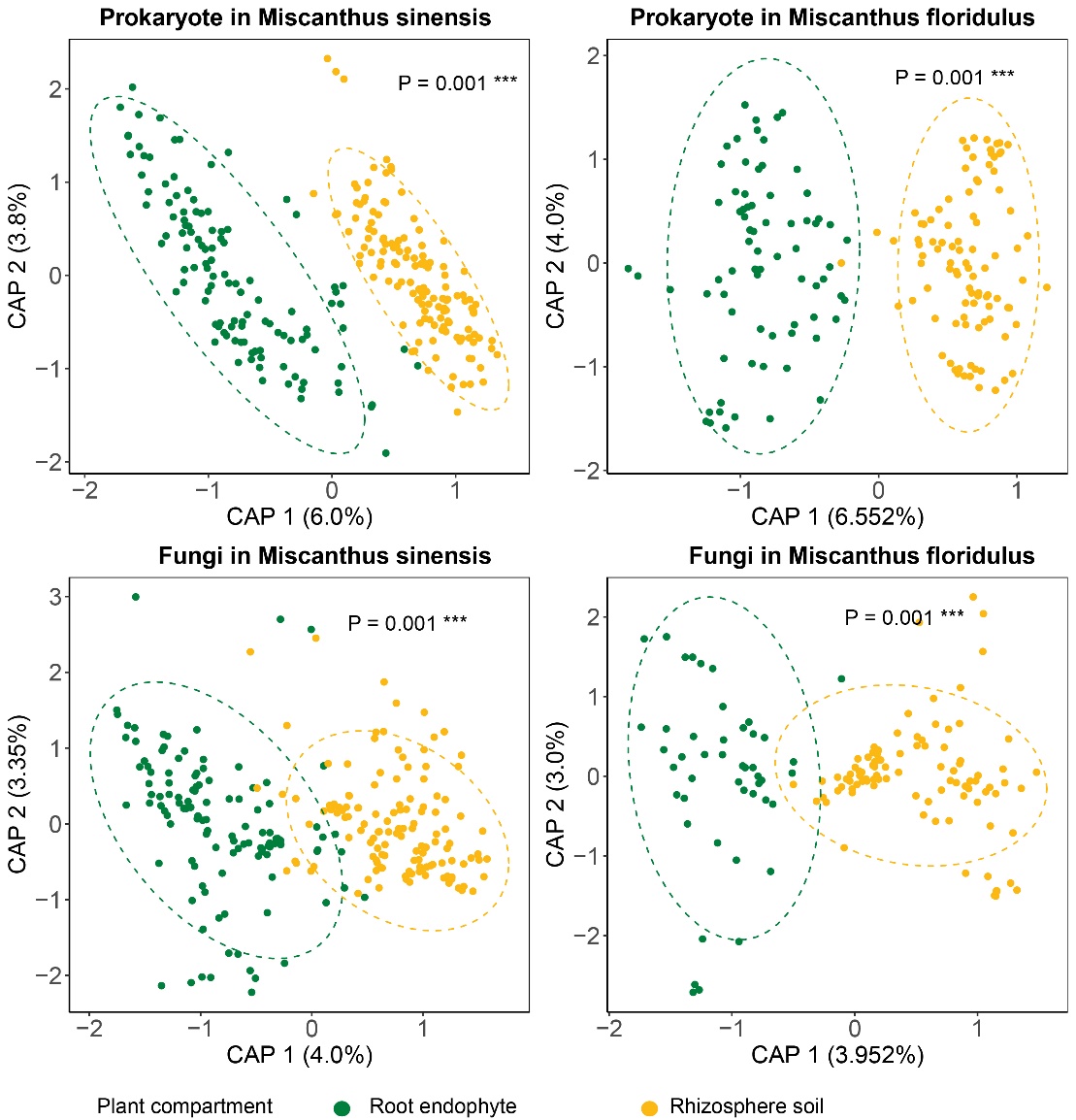


**Figure S6. Partial Canonical analysis of Principal Coordinates (CAP) of rhizosphere soil and root endophytic prokaryotic and fungal communities in *M. sinensis* and *M. floridulus*.** The partial CAP ordinations using Bray–Curtis distance were constrained to the root compartment while controlling for effect of site. Axes report the proportions of total variation explained by the constrained axes**.** Significance of prokaryotic and fungal community dissimilarities among different root compartments are based on using PERMANOVA tests.

**Figure S7**


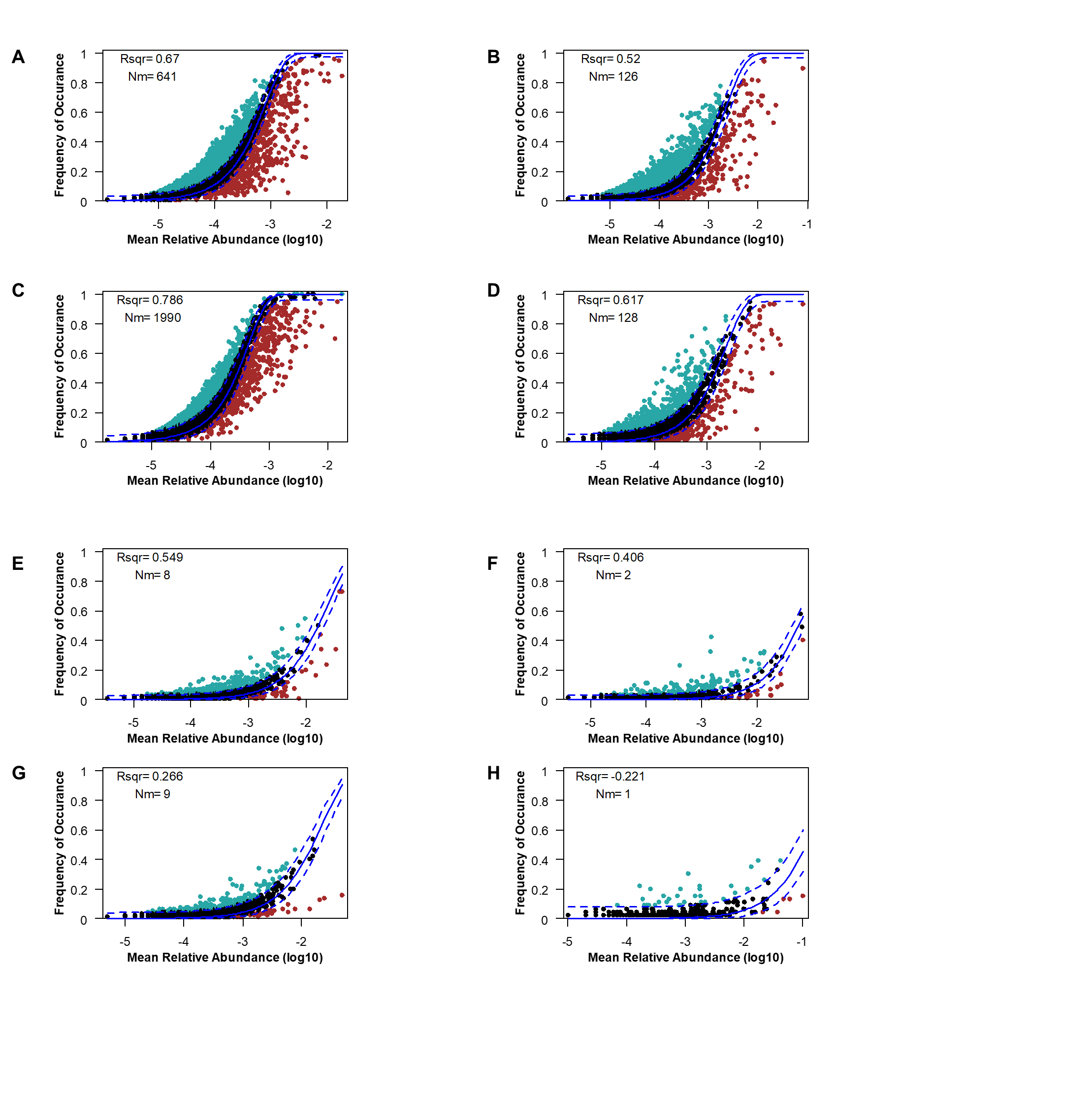


**Figure S7.** **Fit of the neutral community model (NCM) of community assembly.** The predicted occurrence frequencies for *M. sinensis* rhizosphere soil prokaryote (A), *M. sinensis* root endophytic prokaryote (B), *M. floridulus* rhizosphere soil prokaryote (C), *M. floridulus* root endophytic prokaryote (D), *M. sinensis* rhizosphere soil fungi (E), *M. sinensis* root endophytic fungi (F), *M. floridulus* rhizosphere soil fungi (G), and *M. floridulus* root endophytic fungi (H). The solid blue lines indicate the best fit to the NCM, and the dashed blue lines represent 95% confidence intervals around the model prediction. OTUs that occur more or less frequently than predicted by the NCM are shown in different colors. Nm indicates the microbial community size times immigration, R^2^ indicates the fit to this model.

**Figure S8**


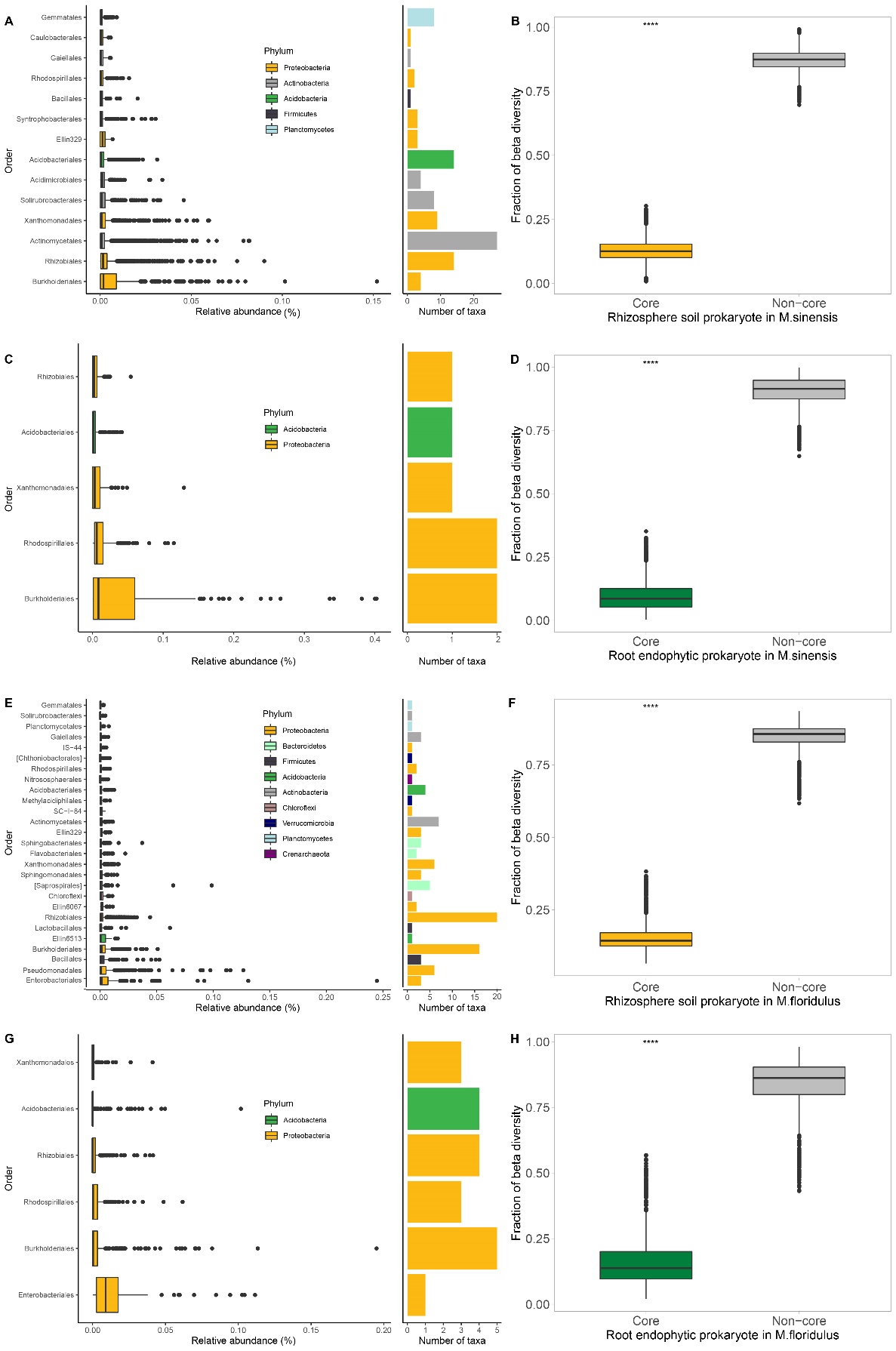


**Figure S8. Core prokaryote in the rhizosphere soil and root endophyte of *M. sinensis* and *M. floridulus*.** Relative abundance of core prokaryotic taxa is represented as boxplots, grouped by order and number of taxa there in rhizosphere soil (A) and root endophyte (C) of *M. sinensis* and in rhizosphere soil (E) and root endophyte (G) of *M. floridulus*. Panels A, C, E, and G are color-coded by phylum. Contributions of the core taxa to changes in beta diversity in each compartment (B, D, F, H).
